# Beef Suppositories

**Published:** 1881-01

**Authors:** James I. Tucker


### NOTES FROM PRIVATE PRACTICE.

### ARTICLE IX.

*Beef Suppositories.*

Though the rectum is, strictly speaking, an excretory organ, it may nevertheless, by virtue of its absorbing power, take the place of the stomach and small intestine in the ingestion of medicinal and alimentary agents. Dupuytren used to say that, owing to the absence of digestion the agent passes more directly, more purely and more surely to its destination from the rectum than it does when taken by the stomach. Hence the speedy efficacy of chloral in mania and the vomiting of pregnancy; of opium and ipecac in dysentery, etc. With this fact in view I have lately used Johnston's or Liebig's beef extract incorporated with cocoa butter in the form of suppository to support life in chronic gastric disorders, adynamic diseases and all cases where the administration of food by the ordinary channel was impossible. The beef is easily combined with the butter, or to save time or for other reasons, the hollow suppositories may be used. The advantage of these suppositories over the beef *injection* will immediately commend itself. DR. JAMES I. TUCKER.

